# Interplay of Oxidative Stress, Autophagy, and Rubicon in Ovarian Follicle Dynamics: Orchestrating Ovarian Aging

**DOI:** 10.3390/antiox14080919

**Published:** 2025-07-27

**Authors:** Kiyotaka Yamada, Masami Ito, Haruka Nunomura, Takashi Nishigori, Atsushi Furuta, Mihoko Yoshida, Akemi Yamaki, Kanto Shozu, Ippei Yasuda, Sayaka Tsuda, Tomoko Shima, Akitoshi Nakashima

**Affiliations:** Department of Obstetrics and Gynecology, University of Toyama, 2630 Sugitani, Toyama 930-0194, Japan; kytkymd1@med.u-toyama.ac.jp (K.Y.); msmito@med.u-toyama.ac.jp (M.I.); nunoharu@med.u-toyama.ac.jp (H.N.); tnishigo@med.u-toyama.ac.jp (T.N.); s0950074@med.u-toyama.ac.jp (A.F.); mihoko.yoshida@med.tonami.toyama.jp (M.Y.); au@med.u-toyama.ac.jp (A.Y.); kshozu@med.u-toyama.ac.jp (K.S.); ippeiy@med.u-toyama.ac.jp (I.Y.); syk3326@med.u-toyama.ac.jp (S.T.); shitoko@med.u-toyama.ac.jp (T.S.)

**Keywords:** autophagy, granulosa cells, ovarian aging, oxidative stress, rubicon

## Abstract

Organ functions generally decline with age, but the ovary is a prototypical organ that undergoes functional loss over time. Autophagy plays a crucial role in maintaining organ homeostasis, and age-related upregulation of the autophagy inhibitor protein, Rubicon, has been linked to cellular and tissue dysfunction. This review describes how granulosa cell autophagy supports follicular growth and oocyte selection and maturation by regulating cellular energy metabolism and protein quality control. We then introduce the role of selective autophagy, including mitophagy or lipophagy, in steroidogenesis and cellular remodeling during luteinization. In aged ovaries, Rubicon accumulation suppresses autophagic flux, leading to diminished oxidative-stress resilience and enhanced DNA damage. Moreover, impaired autophagy drives the accumulation of ATP citrate lyase, which correlates with poor oocyte quality and reduced ovarian reserve. Following fertilization, oocytes further upregulate autophagy to provide the energy required for blastocyst transition. Conversely, in infertility-related disorders, such as premature ovarian insufficiency, endometriosis, and polycystic ovary syndrome, either deficient or excessive autophagy contributes to disease pathogenesis. Both autophagy inhibitors (e.g., Rubicon) and activators (e.g., Beclin1) could be emerging as promising biomarkers for assessing ovarian autophagy status. Therapeutically, Rubicon inhibition by trehalose in aged ovaries and autophagy suppression by agents such as hydroxychloroquine in polycystic ovary syndrome and endometriosis hold potential. Establishing robust methods to evaluate ovarian autophagy will be essential for translating these insights into targeted treatments.

## 1. Introduction

Germ cells (oocytes) in the mammalian ovary differ fundamentally from spermatogonial stem cells in that their proliferation ceases during fetal life and thereafter only declines. The average age at menopause is approximately 50 years, at which time the resting oocyte pool has decreased to roughly 1000 cells; in contrast, there are about two million oocytes at birth, which decrease to approximately 0.3 million by puberty [1]. Like dogs, horses, and cattle, humans exhibit spontaneous ovulation independent of copulation, selecting a single dominant follicle while the remaining follicles undergo atresia. Mono-ovulation is orchestrated by intrafollicular intercellular communication, the precise regulation of hormone receptor expression, and programmed cell death processes, all of which likely underlie the age-associated deterioration of oocyte quality. Within this communication network, granulosa cells (GCs) constitute the principal somatic component of the follicle, playing indispensable roles in folliculogenesis and oocyte maturation [2]. Elucidating the molecular mechanisms governing GCs development and function is essential for deepening our understanding of follicular dynamics, advancing infertility treatment strategies, overcoming ovarian pathologies, and improving assisted reproductive technologies. So far, we have demonstrated that impaired autophagy contributes to placental insufficiency and the development of preeclampsia [3,4,5,6], and more recently that dysregulated autophagy is implicated in reproductive disorders such as endometriosis and ovarian dysfunction [7,8]. In this review, we provide an overview of the functions of autophagy, explore its roles in follicular development and hormone production mechanisms, and discuss age-related physiological changes in the ovary, particularly with a focus on Rubicon. We further examine ovarian dysfunction-related diseases in the context of autophagy and conclude by outlining potential future directions for diagnostic and therapeutic interventions.

## 2. Autophagy

Autophagy, “self-eating”, is an evolutionarily conserved, lysosome-mediated intracellular degradation pathway that targets superfluous or damaged proteins and organelles for breakdown and recycling. This process is indispensable for cellular homeostasis, enabling survival under nutrient deprivation, regulating energy metabolism, and maintaining protein quality control. In the early 2000s, Tamotsu Yoshimori and colleagues identified Microtubule Associated Protein 1 Light Chain 3 (MAP1LC3, commonly known as LC3), the mammalian homolog of yeast Atg8, as a marker of autophagosomes, and this discovery catalyzed the rapid elucidation of autophagy’s molecular mechanisms [9]. Since then, autophagy has been linked to the pathogenesis of cancer, neurodegeneration, metabolic and cardiovascular diseases, and aging. Reflecting its central role in health and longevity, metabolic sensors such as AMP-activated protein kinase (AMPK) and mechanistic target of rapamycin (mTOR) tightly regulate autophagic activity, thereby positioning autophagy as a key longevity control mechanism [10,11,12].

**Figure 1:**
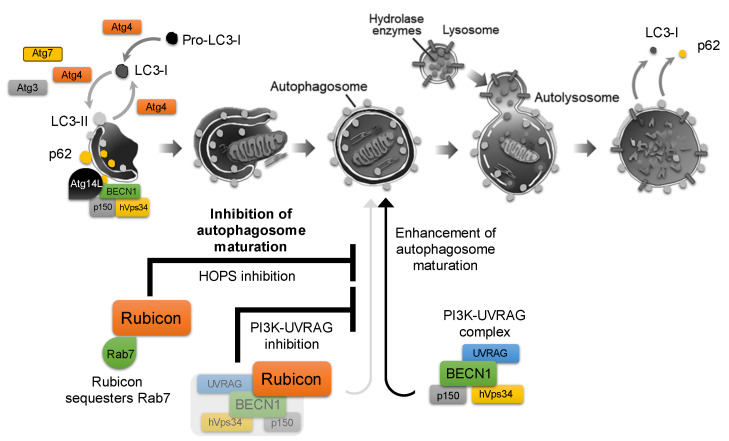
**Role of Rubicon in the autophagy pathway.** Autophagy is initiated by phagophore nucleation and elongation to form the double-membrane autophagosome, which then fuses with lysosomes to generate the autolysosome, where enclosed cargo is degraded. Following cargo degradation, p62 dissociates from the isolation membrane, and LC3-II is delipidated to LC3-I and released from the membrane. The class III PI3K complex, which is composed of Beclin-1 (BECN1), hVps34, and p150, acts as an autophagosome-formation driver when bound to Atg14L, whereas its association with UVRAG promotes autophagosome maturation. Rubicon binds to the PI3K complex and directly interacts with Rab7, thereby inhibiting the Rab7–HOPS (homotypic fusion and protein sorting) machinery and suppressing autophagosome formation, which contributes to overall autophagy inhibition.

ATG4 is involved in both the activation (phosphatidyl-ethanolamine (PE) conjugation) and the subsequent inactivation (deconjugation) of ATG8/LC3. As starvation is a known inducer of autophagy, starvation-induced increases in reactive oxygen species (ROS) can inhibit ATG4′s deconjugating activity. This allows ATG8–PE to persist on autophagosome membranes, thereby promoting autophagy. However, during prolonged starvation, ATG4’s priming activity is also suppressed, ultimately leading to autophagy inhibition [13]. Indeed, ATG4 has been reported to play a critical role in facilitating the conversion of LC3-I to LC3-II in various reproduction-related cells [6]. This redox-sensitive regulation of ATG4 exemplifies how autophagic flux is finely tuned by cellular stress signals. Autophagy is thus controlled by a balance of stimulatory and inhibitory factors, although far more activators have been identified than inhibitors. Indeed, over 40 autophagy-activating factors are known, whereas comparatively few endogenous inhibitors have been characterized. Among these, the most prominent is Run domain Beclin1-interacting and cysteine-rich containing protein (Rubicon), which was first identified in 2009 as a binding partner of Beclin1 (BECN1) that negatively regulates autophagic flux by blocking autophagosome–lysosome fusion [14,15]. It has recently been recognized as one of the few known autophagy inhibitors. Rubicon associates with the class III phosphatidylinositol 3-kinase (PI3K) complex, including hVps34/p150/BECN1, via its RUN and cysteine-rich domains. Knockdown of Rubicon enhances autophagosome formation by relieving Rubicon-mediated inhibition of the PI3K3–UVRAG complex and facilitating activation of the Rab7–HOPS (Homotypic fusion and Protein Sorting) pathway, thereby de-repressing autophagosome maturation and fusion and promoting overall autophagic flux [11] (Figure 1).

Nakamura et al. demonstrated that Rubicon expression rises with age in *C. elegans*, Drosophila, and mice, correlating with suppressed autophagy, organ dysfunction, and reduced lifespan [16]. In the reproductive system, Yamamuro et al. showed that Rubicon deletion in Sertoli cells leads to excessive autophagic degradation of the transcription factor GATA binding protein 4 (GATA4), resulting in impaired spermatogenesis [17]. Similarly, we have reported that age-dependent Rubicon accumulation in ovaries diminishes oxidative stress resistance and promotes DNA damage [8]. Thus, Rubicon functions not merely as an autophagy inhibitor but as an integrative regulator of aging, stress response, and reproductive function. In the sections that follow, we review the roles of Rubicon and other autophagy regulators in ovarian aging and infertility, and discuss the potential for autophagy-targeted therapies in reproductive medicine.

## 3. Role of Autophagy in Granulosa Cells Supporting Follicular Development: Mechanisms of Cell Survival and Quality Control

Follicular development proceeds through both gonadotropin-independent and -dependent phases. Primordial through primary follicles grow over approximately 120 days without gonadotropin stimulation, whereas secondary follicles ≤2 mm in diameter remain follicle-stimulating hormone (FSH)-responsive but at low sensitivity and take ~70 days to develop further. Once follicles reach 2–5 mm, they become highly gonadotropin-sensitive and, in response to cyclical gonadotropin fluctuations, rapidly mature into Graafian follicles within ~14–20 days [18]. During the process, GCs are the principal somatic component of the ovarian follicle and play indispensable roles in folliculogenesis and oocyte maturation [2]. After puberty, FSH drives the proliferation of flat precursor GCs into a multilayered cuboidal epithelium [19]. Within these cells, FSH upregulates aromatase activity, converting theca-derived androgens into estradiol, which in turn feeds back to further stimulate GCs proliferation [2]. GCs also secrete inhibin (INH), which negatively regulates pituitary FSH secretion to fine-tune follicle selection and growth with estradiol [20]. During the antral stage, GCs differentiate into cumulus and mural subpopulations that fulfill distinct functions, under the influence of paracrine factors such as epidermal growth factor (EGF) and bone morphogenetic protein (BMP)-7 [21]. Intrafollicular communication via EGFR ligands, BMPs, and gap junctions further coordinates growth and suppresses apoptosis across the follicular hierarchy [19,22].

**Figure 2:**
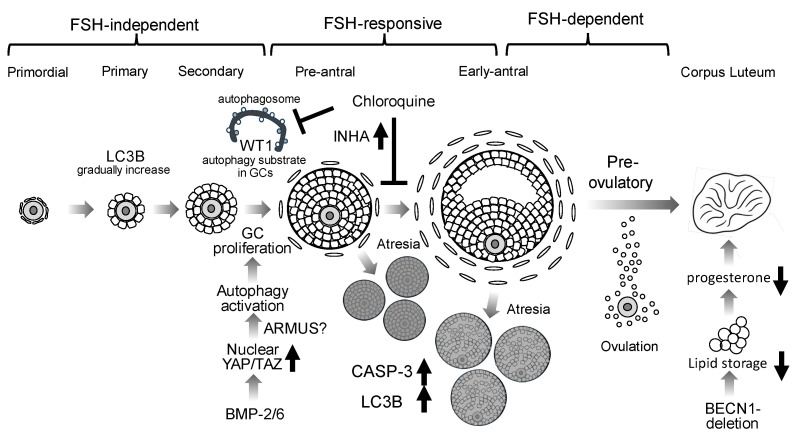
**Putative roles of autophagy in folliculogenesis.** Autophagy involvement in granulosa cells (GCs) during follicular development is depicted. LC3B is expressed from primary through antral follicles. BMP2 and BMP6 suppress the Hippo pathway via nuclear translocation of YAP/TAZ, resulting in activation of autophagy and promoting GC proliferation. The accumulation of WT1, an autophagy substrate, inhibits GC differentiation: Chloroquine, an autophagy inhibitor, inhibits WT1 degradation in GCs. Chloroquine also elevates inhibin A (INHA) levels, thereby inhibiting GC differentiation. During follicle selection, atretic follicles (Atresia) exhibit increased caspase-3 (CASP-3) and LC3B expressions. In GC-specific BECN1 knockout mice, luteal neutral lipid stores are markedly reduced, resulting in diminished progesterone production.

Figure 2 illustrates the involvement of autophagy at each stage of folliculogenesis (Figure 2). Autophagy is highly active in GCs: LC3B expression peaks from the primary to large antral stages, with pronounced upregulation in GCs [23]. Key transcription factors Wilms tumor 1 (WT1) and forkhead box O1 (FOXO1) normally restrain GCs differentiation, and WT1 itself, which is mainly expressed in GCs until the secondary follicular stage, is a substrate for autophagic degradation [24]. siRNA-mediated knockdown of ATG5 or BECN1 in KGN cells, a granulosa cell tumor–derived cell line, attenuates autophagy, reduces expression of GCs’ differentiation markers, such as cytochrome P450 family 19 subfamily A member 1 (CYP19A1), FSHR, and steroidogenic acute regulatory protein (StAR), and lowers estradiol synthesis [24]. In GCs from biochemical POI patients, p62/SQSTM1 accumulation indicates impaired autophagy, and postmenopausal human ovaries exhibit Rubicon accumulation, further linking autophagy suppression to ovarian aging [8]. Interestingly, in Sertoli cells, which also produce INH, FSHR-positive cells co-expressing high levels of WT1 and GATA4 show robust phagocytic activity to maintain local immune privilege. Meanwhile, Rubicon knockout in mouse testes hyperactivates autophagy, accompanied by GATA4 downregulation, resulting in reduced testis weight, litter size, and sperm motility [17,25]. These findings suggest that Rubicon protects Sertoli cell functions in male fertility by regulating autophagy, a process inhibited by androgen.

FSH also directly activates autophagy in GCs: FSH treatment increases HIF-1α and induces BECN1 expression, thereby promoting autophagic flux [26]. Through the PTEN-induced kinase 1 (PINK1)–parkin RBR E3 ubiquitin protein ligase (PRKN) pathway, FSH maintains mitochondrial membrane potential and triggers mitophagy; pharmacologic inhibition of this pathway impairs follicular growth [26]. Lactate similarly stimulates GC autophagy via the HIF-1α/BNIP3/Beclin1 axis [27].

Bidirectional crosstalk between oocytes and GCs is central to follicle development. GCs supply metabolic substrates and regulatory signals to the oocyte via gap junctions and transzonal projections [2], while oocyte-derived growth differentiation factor (GDF)-9 and BMP-15 promote GC proliferation, differentiation, and follicular cavity formation [19]. BMP-15 specifically induces autophagy and suppresses apoptosis in GCs [28]. In mice deficient of GDF-9, which are infertile due to arrest at the primary follicle stage [21], early exposure to fumonisin B1 causes transgenerational repression of GDF-9 and autophagy in F1 ovaries, followed by compensatory autophagy activation in F2, implicating GDF-9–mediated autophagy in the integrity of ovarian function [29]. Collectively, these data underscore how autophagy in GCs integrates hormonal, metabolic, and paracrine signals to orchestrate follicular growth and oocyte quality.

We have previously identified BMP-2, abundantly expressed in GCs, as an FSH-independent promoter of early follicle growth: BMP-2 drives YAP1 nuclear translocation, thereby increasing GC proliferation and supporting FSH-independent folliculogenesis [30]. Nutrient deprivation activates autophagic flux via YAP/TAZ signaling: nuclear YAP induces transcription of the Rab-GAP family member protein TBC1 domain family member 2 (also known as Armus), which facilitates autophagosome–lysosome fusion [31] (Figure 2). BMP-6 also engages the Hippo pathway to promote TAZ nuclear entry and upregulates genes involved in cell proliferation and angiogenesis, further enhancing follicle growth [32]. Conversely, high cell density in non-transformed cells sequesters YAP/TAZ in the cytoplasm, suppressing autophagy [33]. Thus, Hippo-mediated autophagy inhibition may occur in Graafian-stage granulosa cells. Given the intense proliferation and resulting high cell density of granulosa cells in Graafian follicles, it is plausible that Hippo-pathway activation contributes to autophagy suppression in this context.

**Figure 3:**
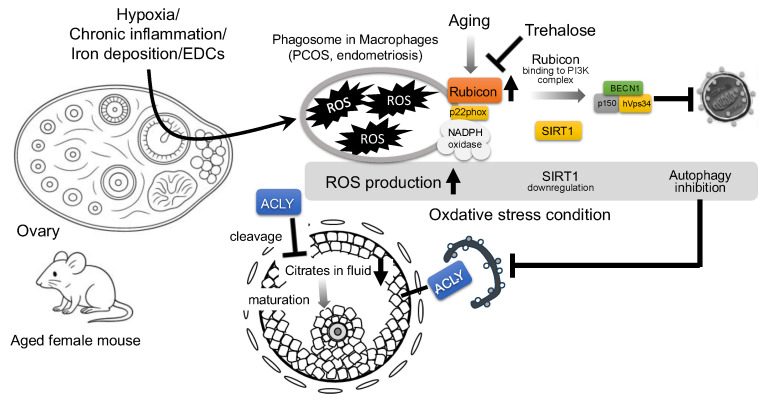
**Proposed mechanism of Rubicon-mediated autophagy suppression and follicular growth impairment in aged mouse ovary.** Environmental stressors, such as hypoxia, chronic inflammation, iron deposition, and endocrine-disrupting chemicals (EDCs), increase reactive oxygen species (ROS) in granulosa cells (GCs). In aged ovaries, the autophagy inhibitor, Rubicon, is upregulated; independently of the autophagy pathway, Rubicon binds the NADPH oxidase subunit p22*phox* in macrophages to further elevate ROS. Elevated ROS downregulates Sirtuin 1 (SIRT1) expression, thereby promoting Rubicon association with the class III PI3K complex (BECN1–hVps34–p150) and suppressing autophagy. Trehalose has been shown to decrease Rubicon expression and may therefore attenuate Rubicon-mediated autophagy inhibition. Concurrently, autophagy inhibition stabilizes ATP citrate lyase (ACLY), which is normally degraded by autophagy, leading to its pathological accumulation; ACLY buildup in GCs depletes follicular citrate, resulting in the impairment of oocyte maturation.

Age-related oxidative stress, accompanied by Rubicon accumulation, further impacts ovarian aging. Independent of its autophagy function, Rubicon binds the p22phox subunit of the NADPH oxidase complex, promoting the production of ROS, TNF-α, and IL-6 in macrophages [34]. Oxidative stress also suppresses Sirtuin 1 (SIRT1) expression, a key regulator of cellular stress in granulosa cells [35]. SIRT1 loss facilitates the binding of PI3K complex to Rubicon, thereby inhibiting autophagosome maturation [36] (Figure 3). Long exposure to hydrogen peroxide (H_2_O_2_) did not significantly change autophagic flux in the human granulosa cell line, but trehalose treatment reduced H_2_O_2_-induced DNA damage in GCs by lowering Rubicon levels without altering BECN1. Not only trehalose (in human GCs) but also quercetin (in rat GCs) enhance resistance to H_2_O_2_-induced cellular damage by activating autophagy [8,37]. In this respect, the BECN1–Rubicon complex likely plays a central role in oxidative stress–induced cytotoxicity in granulosa cells; disruption of this complex mitigates cell damage.

## 4. Crosstalk Between Steroidogenesis and Autophagy in Granulosa Cells

FSH–driven estrogen production in ovarian GCs is tightly regulated by pituitary gonadotropins. The binding of FSH to its receptor, FSHR, activates the Gs protein, stimulating adenylyl cyclase to raise intracellular cAMP, thereby triggering protein kinase A (PKA) signaling [38]. This cascade induces transcription of the aromatase enzyme, CYP19A1, which converts theca-derived androgens into estradiol [39]. Concurrently, expression of StAR and cholesterol side-chain cleavage enzyme, CYP11A1, is upregulated, enhancing pregnenolone synthesis from cholesterol [40]. In mice, FSH increases the number of pre-ovulatory and antral follicles, but co-treatment with chloroquine, an autophagy inhibitor, suppresses follicular growth. Under chloroquine exposure, transcription of the progesterone biosynthetic enzyme hydroxy-delta-5-steroid dehydrogenase, 3 beta- and steroid delta-isomerase 2 (HSD3B2) is elevated, whereas INHA expression decreases; CYP11A1 and StAR levels remain unchanged [26] (Figure 2).

During the pre-ovulatory LH surge, luteinizing hormone (LH) binds luteinizing hormone/choriogonadotropin receptor (LHCGR) on post-antral GCs, again engaging the cAMP/PKA axis to drive final differentiation, so-called luteinization, and shift steroid output from estradiol to progesterone, a process also involving PKA-independent cAMP signaling [41]. LH stimulation further enhances StAR and CYP11A1 expression and activity, leading to robust progesterone secretion by luteal cells [42].

In GCs-specific BECN1 deletion in ovaries, luteal neutral lipid stores are markedly reduced, resulting in diminished progesterone production; electron microscopy reveals reduced endoplasmic reticulum dilation and fewer whorled membrane structures, consistent with impaired steroidogenic capacity [43] (Figure 2). Human chorionic gonadotropin (hCG) mimics LH action in cultured human luteinized GCs, increasing StAR and HSD3B2 expression while activating lipophagy via LAMP2-mediated lipid droplet–lysosome interactions to further augment progesterone synthesis [44]. In GCs from patients with luteal phase insufficiency, mRNA levels of autophagy-related genes (BECN1, AMBRA1, ATG5, ATG16L1, etc.) are downregulated, and the normal hCG-induced increase in LC3B-II/LC3B-I ratio and BECN1 protein is blunted [44]. Lastly, FSH promotes c-Jun phosphorylation and nuclear translocation in porcine GCs; c-Jun then binds directly to the BCLN1 promoter, driving BCLN1 expression and autophagy activation [45].

## 5. Autophagy in Follicular Atresia and Regulation of Ovarian Reserve: Selective Cell Death Mechanisms

In the mammalian ovary, over 99% of developing follicles are eliminated via apoptosis (follicular atresia) in GCs throughout the female’s postnatal life, resulting in a marked decline in follicle number and ovarian reserve [46,47]. More than half of all oocytes are also eliminated by apoptosis during the transition from fetal life to the neonatal period, a process that establishes the finite pool of primordial follicles present after birth [48]. In mice, germ cell cyst breakdown begins around embryonic day 17.5, and by postnatal day 5, the ovary is populated by individually enclosed primordial follicles. Autophagy is transiently activated during this perinatal cyst breakdown phase to support normal folliculogenesis, in part by clearing ROS. Furthermore, autophagy contributes to continued follicular development by maintaining the expression of key regulators such as GDF-9, which facilitates follicular structure formation and maintenance, and transforming growth factor-β1, which mediates granulosa–oocyte interactions [49].

Although systemic Atg5 knockout mice die within 12 h of birth due to neural defects [50], targeted neuronal expression of GFP-ATG5 under the NSE promoter rescues viability (Atg5^−^/^−^; NSE-Atg5), allowing assessment of adult gonadal phenotypes [51]. At 8–12 weeks, these rescued females display normal-appearing primary, secondary, and tertiary follicles but lack corpus luteum and exhibit numerous atretic follicles, consistent with an ovulation failure. Additionally, uterine abnormalities, defined by a thread-like uterus and absent endometrial glands, disrupted estrous cycling, and reduced serum and pituitary LH/FSH mRNA levels, indicate hypothalamic-pituitary-gonadal axis dysfunction. Similarly, Atg7-deficient ovaries contain no oocytes at postnatal day 1, underscoring autophagy’s essential role during follicle formation [52]. BECN1 heterozygotes also exhibit a ~50% reduction in oocyte number and numerous pyknotic cells, despite BECN1 expression being confined to follicles rather than ovarian cortex or medulla [52]. These reports collectively demonstrate that autophagy is indispensable for gonadal development and maintenance.

Because the primordial follicle pool progressively diminishes after birth and the oocytes within these follicles remain highly susceptible to oxidative stress, acquired factors, including environmental insults and endocrine disorders, can markedly influence the rate of primordial follicle depletion. The antioxidant enzyme, superoxide dismutase 1 (SOD1), counteracts ROS accumulation and helps protect oocytes from oxidative damage [53]. Notably, SOD1 has been identified as a substrate of autophagy in neuronal cells [54]. The pro-autophagic factor, rubicon-like autophagy enhancer (RUBCNL), activates autophagy by competitively binding to UVRAG in place of Rubicon; however, overexpression of RUBCNL in neuronal models paradoxically promotes the aggregation of mutant SOD1 [55]. This raises the possibility that increased levels of Rubicon (which would inhibit autophagy) might facilitate SOD1 turnover under certain conditions. Examining the regulation of SOD1 and the interplay between RUBCNL and Rubicon in ovarian GCs and oocytes could provide new insights into how oxidative stress is managed in the ovary.

From a clinical perspective, understanding the mechanisms of follicular atresia and the regulation of ovarian reserve is essential for developing interventions to preserve fertility, optimize ovarian stimulation protocols, and predict reproductive longevity in patients undergoing assisted reproductive technologies or facing age-related ovarian decline. Mono-ovulation reduces the risk of multiple gestations, a risk for premature delivery, and reduced survival of offspring. Clinically, FSH–Gonadotropin-Releasing Hormone pulse therapy exploits this by transiently lowering circulating FSH to induce monofollicular growth, thereby minimizing ovarian hyperstimulation syndrome and multiple pregnancies. Reduced FSH disrupts survival signaling in GCs, leading to FoxO3a-mediated upregulation of pro-apoptotic factors (e.g., Bim) and selective follicle atresia [46]. Atretic follicles are thus the consequence of targeted apoptosis in GCs, representing a major determinant of diminished ovarian reserve [47]. Conversely, suppression of death in GCs can preserve reserve; for example, NLR family pyrin domain-containing 3 (NLRP3) inflammasome-deficient mice show attenuated age-related follicle loss and sustained Anti-Müllerian Hormone (AMH) levels, indicating ovarian reserve [56]. At the molecular level, cleaved caspase-3 in atretic GCs positively correlates with the autophagy marker, LC3B-II, across multiple mammalian species [23,57,58,59]. In porcine models, downregulation of the m^6A methyltransferase METTL3 and upregulation of the demethylase FTO alpha-ketoglutarate-dependent dioxygenase reduce m^6A modification on unc-51 like autophagy activating kinase 1 (ULK1) mRNA, preventing YTHDF2-mediated degradation and thereby increasing ULK1 expression [58]. Studies comparing GCs from medium-sized versus large antral follicles reveal that medium-follicle GCs exhibit high proliferation, low p-mTOR, and elevated ATG3/ATG7, which indicate activation of autophagy, whereas large-follicle GCs show low proliferation, increased p-ERK, Bax, and caspase-3, which indicate activation of apoptosis [60]. Given that large-follicle GCs will luteinize, these data suggest that autophagy diminishes, and apoptosis increases during luteinization.

Despite LC3B’s prominence as an autophagy marker, LC3B-knockout mice display no overt phenotype or p62 accumulation [61]. In contrast, GABARAP-deficient mice exhibit increased TUNEL-positive cells and elevated pro-apoptotic proteins, such as Bid, Apaf1, and Bax, in mammary tissue, implying that GABARAP uniquely regulates both autophagy and apoptosis [62]. Structural comparisons in *C. elegans* show that LGG-1, a mammalian homolog of LC3, mediates autophagosome formation and substrate degradation, whereas LGG-2, a mammalian homolog of GABARAP, is required for autophagosome expansion, autophagosome–lysosome fusion, and selective autophagy of specific substrates [63]. Moreover, GABARAP, but not LC3B, binds Bcl-2 to inhibit its PE conjugation, thereby modulating autophagy and apoptosis [64]. Taken together, reduced GABARAP may contribute to apoptosis in GCs during follicular atresia.

## 6. Autophagy in Oocyte Maturation and Ovarian Aging: Maintenance of Oocyte Quality and Age-Related Changes

Autophagy is likewise critical for oocyte maturation. BECN1 knockdown in oocytes significantly reduces first polar body extrusion, causing maturation arrest [65]. From germinal vesicles through metaphase II, LC3-II puncta, which indicate autophagosome formation, are readily detected, demonstrating that autophagy is activated throughout oocyte maturation [65]. In unfertilized murine oocytes, autophagy activation extends cellular survival [66], and the LC3-II/LC3-I ratio rises in bovine oocytes subjected to heat stress (41 °C), indicating an adaptive autophagic response to heat shock [67]. Upon fertilization, embryos exhibit a steep increase in LC3 puncta between the one- and four-cell stages, reflecting a surge in autophagic activity [68]. Atg5-deficient zygotes arrest at the 4- to 8-cell stage, resulting in markedly reduced blastocyst formation. Furthermore, autophagy induction in unfertilized oocytes can be triggered by calcium oscillations or mTOR inhibition, implicating these pathways in fertilization-associated autophagy [69]. Biologically, fertilization-induced autophagy likely degrades maternal proteins to supply amino acids critical for early embryogenesis. In *C. elegans*, fertilization triggers selective autophagic clearance of sperm-derived mitochondria, ensuring maternal mitochondrial inheritance, as evidenced by LGG-1 mutants retaining paternal mitochondria and exhibiting developmental arrest [70]. In mice, however, paternal mitochondria are also eliminated by the ubiquitin–proteasome system [71].

In mammals, including humans, ovarian aging leads to both the decline of oocyte number and oocyte competence, contributing to rising infertility rates and embryonic defects [72]. Aged oocytes accumulate oxidative damage [73]; pronounced protein and lipid peroxidation, as well as mitochondrial dysfunction, already begin at the primordial follicle stage in aged oocytes [74]. Mitochondria serve as the principal energy source but also the major generator of ROS in oocytes [72]. In aged ovaries, diminished antioxidant capacity exacerbates oxidative stress and mitochondrial dysfunction, which in turn deranges oocyte bioenergetics: aged oocytes have increased reliance on glycolysis due to depletion of essential metabolites such as NAD^+^ [74]. Follicular fluid, which is secreted from theca capillaries and GCs, exhibits age-associated metabolomic shifts: increases in threonine, trans-4-hydroxyproline, histidine, N^2^,N^2^-dimethylguanosine, and γ-glutamylvaline correlate with ovarian senescence and diminished oocyte quality, likely reflecting enhanced glutathione recycling under oxidative stress [75]. Rubicon, an autophagy suppressor whose knockout extends organismal lifespan via systemic autophagy activation [16] and whose expression is elevated in postmenopausal versus premenopausal ovaries [8], likely mediates ovarian autophagy suppression; in turn, autophagy inhibition in aged granulosa cells stabilizes the normally autophagy-degraded enzyme ATP citrate lyase (ACLY), causing its pathological accumulation and impaired oocyte maturation due to citrate reduction [76], which suggests that Rubicon-mediated autophagy suppression in these cells could underlie the age-related decline in oocyte quality (Figure 3). Although Rubicon is essential for sustaining testicular function, its increased expression in the ovary appears to contribute to ovarian dysfunction.

**Figure 4:**
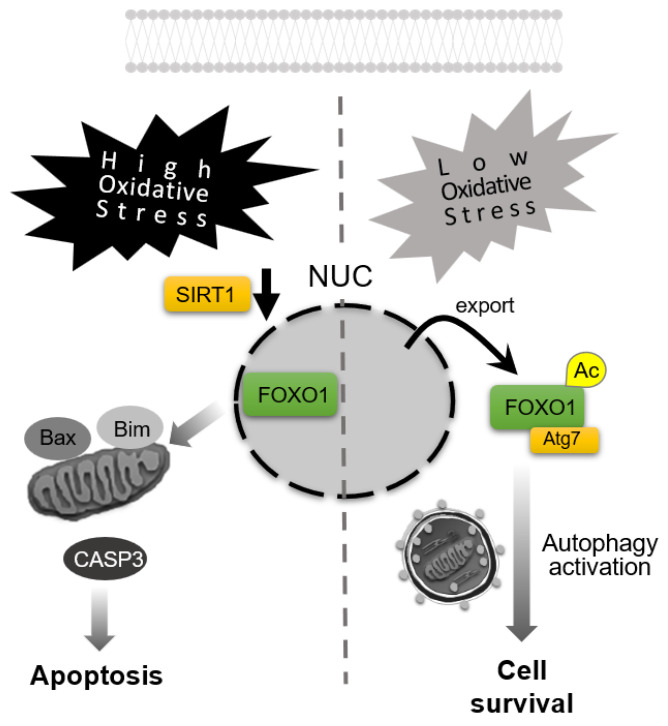
**Apoptosis–autophagy switching in response to oxidative stress levels.** High-level oxidative stress downregulates Sirtuin 1 (SIRT1) expression, resulting in forkhead box O1 (FOXO1) retention in the nucleus and induction of apoptosis, accompanied by the activation of caspase-3 (CASP3). In contrast, under low-level oxidative stress, acetylated FOXO1 (Ac-FOXO1) translocates to the cytoplasm, binds ATG7 to activate autophagy for promoting cell survival. NUC: nuclei.

Consistent with these findings, studies in aged pigs have identified molecular changes linking autophagy, oxidative stress, and antioxidant defenses. In the ovaries of older pigs, markers of impaired autophagy are observed alongside reduced Sirt1 and SOD1 expression, whereas the lipid phosphatase, phospholipid phosphatase 3 (PLPP3), is significantly upregulated [77]. In granulosa cell cultures, overexpression of PLPP3 decreases Sirt1 and antioxidant enzymes, catalase and SOD1, whose knockout in mice blocks normal folliculogenesis [78], accompanied by elevated ROS levels [77]. Thus, PLPP3 accumulation in aging ovaries may exacerbate oxidative stress by downregulating Sirt1 and antioxidant defenses. Sirt1, via deacetylation of FoxO1, enhances FoxO1’s activity on longevity and metabolic genes. Intriguingly, FoxO1 can act as a redox-sensitive switch in GCs: under low oxidative stress, acetylated FoxO1 translocates to the cytoplasm and binds Atg7 to promote autophagy and maintain homeostasis, whereas high oxidative stress retains FoxO1 in the nucleus, where it upregulates pro-apoptotic targets, in porcine endometrial cells [79] (Figure 4). Melatonin, a pleiotropic indoleamine hormone produced in the ovary [80], has been shown to counteract oxidative ovarian aging by increasing Sirt1 levels and activating mitophagy, thereby reducing ROS in GCs [81].

Selective autophagic removal of damaged mitochondria, mitophagy, is thus vital. In porcine oocytes, oxidative stress induces PINK1, PRKN, and LC3-II upregulation, whereas α-tocopherol attenuates the stress and restores PINK1/PRKN-mediated mitophagy [82]. Supplementation of spermidine, a polyamine whose levels halve in old mice aged 52–56 weeks, via drinking water rescues follicular growth and oocyte maturation, resulting in enhancing early embryo development and fetal yield by improving mitochondrial function through mitophagy induction [83].

In addition to disrupting metabolism, chronic oxidative stress in aging oocytes leads to accumulating DNA damage, particularly DNA double-strand breaks (DSBs). Repair of such lesions depends on the Ataxia-telangiectasia mutated (ATM)–BRCA1 DNA repair-associated (BRCA1)/2 pathway, and impaired DNA repair accelerates follicular attrition, potentially precipitating premature ovarian insufficiency (POI, also termed premature ovarian failure). Clinically, women carrying BRCA1 mutations, which are detected in women with hereditary breast and ovarian cancer syndrome, tend to have decreased ovarian reserve, evidenced by lower AMH levels and reduced oocyte counts. In mice, Atm functions upstream of Brca1 in the DNA damage response; correspondingly, Brca1-knockout females exhibit a marked reduction in follicle numbers [84], and even Atm^+^/^−^ females show significantly fewer primordial, primary, and total follicles than wild-type controls [85]. Notably, the ATM–Chk2 pathway can activate autophagy to facilitate the clearance of DNA damage, whereas autophagy inhibition exacerbates genomic damage [86]. These observations suggest that the inhibition of the ATM/BRCA1/Chk2-mediated autophagy pathway compounds DNA damage accumulation in aging oocytes. Furthermore, epigenetic alterations have been linked to autophagy downregulation in ovarian aging. In a 24-month-old rat model as an aged phenotype, ovarian mRNA and protein levels of several core autophagy genes (Atg5, Atg12, Atg16L, Beclin1, and LC3B) were significantly reduced relative to young controls. This was accompanied by pronounced hypermethylation of the promoter regions of Atg5 and LC3B, and increased ovarian expression of the de novo DNA methyltransferases DNA methyltransferase (DNMT)3A and DNMT3B [87]. Thus, age-related CpG methylation of autophagy gene promoters contributes to diminished autophagic activity in the ovary.

**Figure 5:**
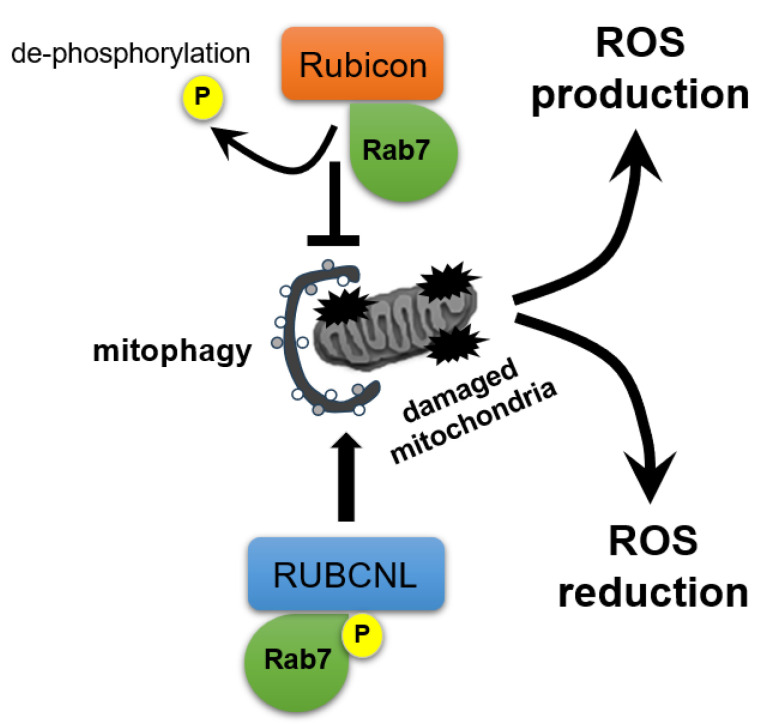
**Switching between Rubicon and RUBCNL in response to mitochondrial damage for ROS regulation.** Rubicon regulates mitochondrial homeostasis: in the basal state, dephosphorylated RAB7A associates with Rubicon, suppressing PRKN-dependent mitophagy and promoting ROS generation; whereas upon mitochondrial depolarization, phosphorylation of RAB7A at Ser72 (P) displaces Rubicon and recruits RubiconL (RUBCNL), enhancing mitophagy to decrease ROS levels.

Collectively, factors governing oocyte maturation and quality, including oxidative stress, mitochondrial health, and the follicular microenvironment, undergo multifaceted, interrelated changes with age, culminating in diminished developmental competence. Both macroautophagy and mitophagy maintain these functions, whereas age-dependent Rubicon upregulation could impair them [8]. As for the mechanism by which Rubicon regulates homeostasis in mitochondria, dephosphorylated RAB7A associates with Rubicon to suppress PRKN-dependent mitophagy in the basal state; whereas upon mitochondrial depolarization, phosphorylation of RAB7A at Ser72 displaces Rubicon and recruits RUBCNL, thereby promoting mitophagy [88] (Figure 5). Additionally, overexpressed Rubicon enhances TNF-α, IL-6, and ROS production in macrophages [34], supporting its role in ovarian aging.

## 7. Involvement of Autophagy in Infertility-Related Disorders: Pathogenic Mechanisms in POI, Endometriosis, PCOS, and Iatrogenic Menopause with Implications for Oncofertility

Ovarian infertility arises from intrinsic ovarian dysfunction, with key associated disorders including POI, polycystic ovary syndrome (PCOS), endometriosis, and iatrogenic ovarian insufficiency. In each case, molecular and genetic perturbations within the ovary impair folliculogenesis and deplete ovarian reserve, leading to infertility. Recent advances suggest that autophagy plays a significant role in these disease processes, potentially offering novel therapeutic strategies beyond conventional hormone-based treatments. However, despite growing recognition of autophagy’s importance in ovarian physiology and pathology, its precise roles remain incompletely understood. This is largely due to the technical challenges involved in accurately assessing autophagic activity [89]. Such difficulties have hindered efforts to elucidate how autophagy contributes to the pathogenesis of these disorders. In this section, we aim to provide a comprehensive overview of the current understanding of autophagy status in each of these infertility-related disorders.

POI, which is defined by follicular depletion before age 40, is a major cause of female infertility [90]. Mutations in oocyte-derived growth factors such as BMP15 and GDF9 have been identified in POI patients [90]. The FOXL2 transcription factor is essential for ovarian differentiation and follicular maintenance; its functional deficiency induces the expression of testis-specific genes within the embryonic ovary, thereby disrupting normal ovarian development [91]. Recently, loss-of-function variants in ATG7 (p.Phe403Leu) and ATG9A (p.Arg758Cys) were discovered in POI cohorts [92]. These variants reduce autophagic flux, compromising the energy supply essential for primordial follicle survival, and likely contribute to diminished ovarian reserve. GCs from POI patients exhibit aberrant morphology, reduced proliferation, increased apoptosis, elevated ROS, and marked downregulation of autophagy markers (ATG7, BECN1, LC3B) with concomitant p62 accumulation, indicating heightened cellular stress from autophagy impairment [93]. Central to this mechanism, POI-derived GCs display reduced FOXO3A, which normally binds promoters of autophagy genes such as LC3, BNIP3, and BNIP3L to drive their transcription [94]. In organotypic culture of whole ovaries, AMH, which is produced by growing follicles from the primary to small antral stages, activates FOXO3A and upregulates multiple autophagy-related genes (ULK1, ATG2A, ATG2B, ATG5, BECN1, GABARAPL2, ATG16L1, WIPI2, ZFYVE1), suggesting that age-related AMH decline may reduce ovarian autophagy [95]. As a murine model of POI, Ectopic P-granules 5 autophagy tethering factor (Epg5) deficiency recapitulates POI phenotypes, implicating autophagic WT1 turnover in follicular integrity [96]. Oxidative stress is increasingly recognized in POI pathophysiology. Plasma levels of advanced oxidation protein products (AOPPs) are significantly elevated in women with POI, including those with incipient biochemical POI, compared to age-matched controls [97]. In a rat model, excessive AOPP exposure activated the mTOR pathway via ROS, impairing nuclear translocation of the transcription factor EB (TFEB), a central regulator of autophagy, thereby suppressing autophagy in ovarian GCs [98]. Such autophagic dysfunction may contribute to follicular loss in POI, highlighting TFEB as a promising therapeutic target of trehalose.

PCOS is characterized by arrested small antral follicles that fail to ovulate [99]. In PCOS patients, GC analyses reveal decreased FSHR and CYP11A1 expression alongside increased CYP19A1, StAR, and INHBA, reflecting steroidogenic dysregulation and follicular arrest [99]. The PCOS hormonal milieu, which characterizes elevated LH, AMH, insulin, and androgens with relatively low FSH, likely stimulates autophagy via high AMH in GCs. In letrozole-induced PCOS rats, GCs show BECN1 upregulation and excessive autophagy, recapitulating the oxidative stress and reduced antioxidant defenses observed in humans [100]. Metformin ameliorates PCOS pathology, such as weight gain, glucose intolerance, cystic ovaries, and hyperandrogenemia, by activating PI3K/AKT/mTOR signaling to suppress excess autophagy, thereby reducing oxidative stress, decreasing cystic follicles, and restoring luteinization. Conversely, theca cells from PCOS patients accumulate p62, suggesting a cell-type-specific autophagy suppression [7]. Hyperandrogenism inhibits aromatase in GCs, further disrupting estrogen synthesis and follicle growth; BMP-2 and BMP-4, both potent anti-androgenic factors, protect GCs from metabolic stress by YAP1-mediated autophagy inhibition under high-androgen conditions [101,102].

Endometriosis creates chronic pelvic inflammation that impairs the local ovarian environment, reducing reserve and causing infertility. Surgical removal of endometriotic cysts often worsens ovarian reserve because of AMH decline; similarly, pre-in vitro fertilization (IVF) surgical excision is not routinely recommended to improve live birth rates due to AMH decline [103]. In GCs from endometriosis patients, markers of DNA damage (e.g., 8-OHdG, 4-HNE), inflammatory cytokines (e.g., IL-1β, IL-6), endoplasmic reticulum stress markers (phospho-IRE1, phospho-PERK), and apoptosis are all elevated [104,105,106]. Recent work shows that oxidative stress-induced downregulation of the histone methyltransferase EZH2 upregulates IL1R2, suppressing IL–1β–dependent ovulatory signaling and causing luteinized unruptured follicle syndrome [107]. Autophagy is upregulated in GCs from endometriosis patients, as evidenced by increased BECN1 expression, an elevated LC3-II/LC3-I ratio, and decreased p62 levels [108]. Moreover, follicular fluid progesterone concentrations are higher in these patients, and endometriosis-derived GCs with activated autophagy exhibit increased expression of steroidogenic enzymes (StAR, CYP11A1, HSD3B2), mirroring the progesterone decrease observed in Becn1-deficient ovarian models, implicating an essential role of autophagy in progesterone regulation. As progesterone suppresses autophagy in the mouse uterine endometrium [109], the increase of progesterone in endometrial cysts might play a role in the suppression of autophagy. Finally, FIP200, a scaffold protein essential for autophagosome formation and decidualization, is required for progesterone receptor signaling in endometrial stromal cells, and its loss leads to decidualization failure and infertility [110].

In patients with diminished ovarian reserve and cyclophosphamide (CPA)-treated mice, GCs overexpress ERβ along with autophagy factors such as BECN1, ATG7, and LC3. Mechanistically, ERβ overexpression enhances FOXO3A transcriptional activity via promoter binding, driving autophagy; selective ERβ antagonists reverse follicle loss, normalize serum AMH and FSH, increase oocyte yield, blastocyst formation, and live births, highlighting the dual role of autophagy in therapy [111]. Given these insights, autophagy inhibitors such as hydroxychloroquine may hold promise for correcting pathological hormone environments and preserving fertility.

Chemotherapeutic agents are a common cause of iatrogenic ovarian insufficiency, acting through distinct but overlapping pathways centered on mitochondrial dysfunction and ROS accumulation [112]. Many chemotherapy drugs disrupt mitochondrial homeostasis, leading to oxidative stress and subsequent follicular apoptosis. For example, CPA and cisplatin (CDDP) both induce ROS: CPA’s metabolite acrolein depletes glutathione and causes mitochondrial membrane depolarization, triggering cytochrome c release and caspase-dependent cell death; CDDP causes mitochondrial DNA damage and DNA crosslinking, further amplifying cellular stress responses. Doxorubicin similarly elevates ROS via redox cycling and interference with electron transport, resulting in superoxide and H_2_O_2_ production, mitochondrial fragmentation, DSBs, and rapid follicle depletion. Paclitaxel, while less directly reactive, inhibits the mitochondrial respiratory chain, leading to ATP depletion, ROS buildup, and apoptosis. Clinically, high cumulative CPA exposure correlates strongly with POI risk: one study identified a threshold CPA dose ≥8000 mg/m^2^ that significantly increased the likelihood of permanent amenorrhea, defined as FSH >30 IU/L before age 40 [113]. Platinum agents (e.g., cisplatin, carboplatin) are also gonadotoxic, though POI risk is generally lower and more variable than with alkylating agents. Meanwhile, doxorubicin and paclitaxel, when used alone, carry relatively low POI risk, though combined regimens can amplify gonadotoxicity. These differences are thought to arise from whether a drug can target dormant primordial follicles: alkylating agents like CPA damage DNA independent of the cell cycle and thus can eliminate quiescent oocytes, whereas drugs like doxorubicin and paclitaxel primarily affect actively dividing cells, sparing much of the primordial pool. Notably, Rubicon overexpression could exacerbate CDDP-induced DNA damage and GCs death, underlining a synergy between ROS and DNA damage in chemotherapy toxicity [8]. Paradoxically, reducing Rubicon levels by trehalose may thus help mitigate chemotherapy-induced ovarian damage. These insights underscore the importance of early fertility preservation strategies for young cancer patients receiving highly gonadotoxic regimens.

## 8. Therapeutic Prospects for Autophagy Modulation in Infertility Treatment

Recent efforts to improve IVF outcomes have focused on enhancing oocyte quality. Antioxidant supplementation and hormone replacement therapies aimed at mitigating oxidative stress are under active investigation. As part of this anti-stress strategy, autophagy activators such as trehalose and resveratrol have emerged as promising candidates. However, to rigorously assess their effects on ovarian function and ovulatory capacity, it is first essential to establish reliable metrics for intrafollicular autophagy status. Follicular fluid soluble factors are thought to reflect GC physiology, and among these, the autophagy inhibitor Rubicon has garnered particular attention. Although Rubicon is an intracellular protein, its circulating levels have been linked to myocardial infarction risk [114], suggesting that extracellular Rubicon may arise from cellular secretion. By analogy, Rubicon concentrations in follicular fluid, presumably derived from GCs, could serve as a practical biomarker of autophagy suppression. Because endogenous autophagy inhibitors are few compared to the multitude of activators, focusing on inhibitory markers like Rubicon may simplify diagnostic workflows [11]. Pathologies characterized by increased oxidative stress, such as POI and chronological ovarian aging, are prime candidates for therapies that restore autophagic competence. Conversely, disorders exhibiting excessive autophagy activation (e.g., endometriosis, PCOS) may benefit more from biomarkers of autophagy activators such as Beclin1. Future studies should employ proteomic profiling of autophagy regulators across infertility subtypes to develop tailored diagnostic algorithms.

Building on such diagnostic stratification, targeted therapeutic interventions can be devised. We have shown that trehalose treatment lowers Rubicon levels in GCs, counteracting the age-related upregulation of this inhibitor observed in ovarian tissue [8]. However, the dynamics of Rubicon expression at the level of individual follicles during aging remain to be elucidated, representing a key research priority. In cases of pathological autophagy hyperactivation, autophagy inhibitors become attractive treatment options. For example, hydroxychloroquine, which is routinely used during pregnancy to manage systemic lupus erythematosus [115], could be repurposed to attenuate excessive autophagy in endometriosis or PCOS. By normalizing autophagic flux, such treatment may improve ovulation rates, fertilization potential, blastocyst formation, and live birth outcomes. Well-designed clinical trials will be critical to validate these strategies and integrate autophagy-modulating agents into infertility therapeutics.

## 9. Conclusions

Autophagy plays a central role in maintaining ovarian function by regulating granulosa cell homeostasis, steroidogenesis, and oocyte quality. The age-associated upregulation of the autophagy inhibitor Rubicon suppresses autophagic flux, exacerbates oxidative stress, and contributes to DNA damage and diminished ovarian reserve. Dysregulated autophagy is also implicated in infertility-related disorders such as premature ovarian insufficiency, endometriosis, and PCOS. While therapeutic strategies targeting autophagy, including Rubicon inhibition and autophagy modulation, show promise, their clinical application remains limited by the lack of reliable biomarkers. Therefore, the development of robust and reproducible methods for assessing autophagy status in ovarian tissue and follicular fluid is an urgent priority to enable individualized diagnostics and targeted infertility treatments.

## Data Availability

Not applicable.

## Abbreviations

The following abbreviations are used in this manuscript:

ACLY	ATP citrate lyase
AMH	Anti-Müllerian Hormone
AMPK	AMP-activated protein kinase
AOPPs	Advanced oxidation protein products
ATM	Ataxia-telangiectasia mutated
ATG	Autophagy-related gene
BECN1	Beclin1
BMP	Bone morphogenetic protein
CASP-3	Caspase-3
CDDP	Cisplatin
CPA	Cyclophosphamide
CYP11A1	Cytochrome P450 family 11 subfamily A member 1
CYP19A1	Cytochrome P450 family 19 subfamily A member 1
DNMT	DNA methyltransferase
EDCs	Endocrine-disrupting chemicals
EGF	Epidermal growth factor
Epg5	Ectopic P-granules 5 autophagy tethering factor
ER	Estrogen Receptor
FIP200	FAK family kinase-interacting protein of 200 kDa
FSH	Follicle-stimulating hormone
FSHR	Follicle-stimulating hormone receptor
FOX	Forkhead box
GATA4	GATA binding protein 4
GCs	Granulosa cells
GDF	Growth differentiation factor
GSH	Glutathione
hCG	human chorionic gonadotropin
HIF-1α	Hypoxia-inducible factor 1-alpha
HOPS	Homotypic fusion and Protein Sorting
H_2_O_2_	Hydrogen peroxide
HSD3B2	Hydroxy-delta-5-steroid dehydrogenase, 3 beta- and steroid delta-isomerase 2
IVF	In vitro fertilization
INH	Inhibin
LC3B	Microtubule Associated Protein 1 Light Chain 3 beta
LH	Luteinizing hormone
LHCGR	Luteinizing hormone/choriogonadotropin receptor
METTL3	Methyltransferase-like 3
mTOR	Mechanistic target of rapamycin
NADPH	Nicotinamide adenine dinucleotide phosphate
NLRP3	NLR family pyrin domain containing 3
PINK1	PTEN-induced kinase 1
PKA	Protein kinase A
PCOS	Polycystic ovary syndrome
PE	phosphatidyl-ethanolamine
PI3K	Phosphatidylinositol 3-kinase
PLPP3	Phospholipid phosphatase 3
POI	Premature ovarian insufficiency
PRKN	Parkin RBR E3 ubiquitin protein ligase
ROS	Reactive oxygen species
RUBCNL	Rubicon-like autophagy enhancer
Rubicon	Run domain Beclin1-interacting and cysteine-rich containing protein
SIRT1	Sirtuin 1
SOD1	Superoxide dismutase 1
StAR	Steroidogenic acute regulatory protein
TAZ	Transcriptional coactivator with PDZ-binding motif
TFEB	Transcription factor EB
TNF-a	Tumor necrosis factor alpha
ULK1	Unc-51-like autophagy activating kinase 1
UVRAG	UV radiation resistance-associated gene
WT1	Wilms tumor 1
YAP	Yes-associated protein
